# Pre- and intra-operative prognostic factors of facial nerve function in cerebellopontine angle surgery

**DOI:** 10.1007/s00405-022-07556-8

**Published:** 2022-07-30

**Authors:** Francesco Gazia, Àngela Callejo, Marta Pérez-Grau, Susana Lareo, José Prades, Francesc Roca-Ribas, Emilio Amilibia

**Affiliations:** 1 Department of Otorhinolaryngology, Hospital Universitari Germans Trias i Pujol, Universitat Autònoma de Barcelona (UAB), Carretera del Canyet, Badalona, 08916 Barcelona, Spain; 2 Unit of Otorhinolaryngology, Department of Adult and Development Age Human Pathology “Gaetano Barresi”, University of Messina, Messina, Italy

**Keywords:** Electromyography, Stimulation threshold, Vestibular schwannoma, Muscle action potential, Prognosis, Nerve monitoring, Facial nerve

## Abstract

**Purpose:**

The study assesses whether pre- and intraoperative factors linked to electromyography and direct electrical stimulation (DES) of facial nerve can predict facial nerve function in the short- (12 days) and long-term (1 year) after cerebellopontine angle (CPA) tumor resection.

**Methods:**

157 patients who underwent surgical resection of CPA tumors with facial nerve monitoring. Pre-operative factors (age, tumor size, pure tone average), surgical time and intra-operative parameters regarding facial function, minimum stimulation threshold (MST), compound muscle action potential (CMAP) and the difference between proximal and distal CMAP (DPDC) were evaluated.

**Results:**

A correlation between tumor size, MST, CMAP and facial function in both short and long term was found. A higher grade of immediate facial paralysis corresponded to a higher risk of poor outcome after one year. A postoperative House–Brackmann (HB) score of V or VI was correlated with poor outcome in 88.8% and 93.8% of cases. A risk of HB 3 or more, in the long term, was correlated with a tumor size of 20.2 mm. Using an MST of 0.1 mA, for long-term predictions, sensitivity and specificity were 0.62 (95% CI 0.46–0.75) and 0.73 (95% CI 0.61–0.82), respectively. With a CMAP cut-off < 200 µV, for long-term prediction, sensitivity was 0.73 (95% CI 0.53–0.87) and specificity 0.73 (95% CI 0.55–0.85).

**Conclusion:**

The assessment based on the cut-offs described increases the ability to predict facial function. Improving predictive accuracy enables surgeons to address patients’ expectations and to establish an intervention timeline for planning facial reanimation.

**Supplementary Information:**

The online version contains supplementary material available at 10.1007/s00405-022-07556-8.

## Introduction

Skull base surgery, and in particular the resection of cerebellopontine angle (CPA) tumors, entails a significant risk of impaired facial nerve activity. Patients with surgical removal of CPA tumors with facial nerve monitoring have better nerve functional results, especially those with large tumors [1, 2]. Since the introduction of electromyography (EMG) monitoring of the facial nerve by Delgado in 1979, the proportion of severe postoperative facial dysfunction has dropped from 15 to 59% in the pre-monitoring era to 10–33% [3]. Facial nerve monitoring is indispensable in skull base surgery today. According to recent literature reports, the anatomical conservation rate of the facial nerve in vestibular schwannoma (VS) surgery has improved by up to 86–92%; even so, there are some patients who suffer a facial dysfunction after surgery even though the nerve is conserved [4–6].

One of the most important aims of skull base surgery is the preservation of facial nerve function. Facial palsy is associated with increases in anxiety and depression, and poorer quality of life [7, 8]. The capacity to predict post-operative facial outcome is vital for the counselling between surgeon and patient regarding prognosis, expectations, and the prospects for rehabilitation. Several intraoperative monitoring techniques have been used with the aim of saving and predicting facial nerve activity. However, the clinical application of EMG recordings has not been standardized [9]. The EMG criteria that allow the prediction of facial function are widely accepted, but the lack of standardization of the stimulation parameters has made it impossible to establish which method is the best [10]. The two most frequently employed facial monitoring systems are direct electrical stimulation (DES) and free-running EMG; transcranial facial nerve motor-evoked potentials (FMEPs) have been introduced more recently. DES implies the use of an impulse of constant voltage or constant current to the VII cranial nerve within the CPA through a mono- or bipolar stimulator. The VII cranial nerve function obtained as an effect of an electric impulse is perceived by electrodes which register muscle activity. It is amplified through a loudspeaker to show a compound muscle action potential (CMAP) [11]. Intraoperative EMG monitorization is useful not only in tumor resection and facial nerve preservation, but also for the prognosis of postoperative facial nerve outcome [12, 13]. Several parameters can be used to predict the outcome of facial function. Measurement of the nerve minimum stimulation threshold (MST) near the brainstem is one of the most commonly applied. Postoperative facial nerve outcomes are inversely proportional to the MST measured after tumor removal, near the brainstem; a higher MST indicates a worse outcome for facial function [14].

The purpose of the present study is to retrospectively evaluate whether the intraoperative MST, CMAP and the difference between proximal and distal CMAP from facial monitoring can predict facial nerve function in the short (12 days) and the long term (1 year) after VS resection. We also analyse whether preoperative factors (age, size, tonal audiometry) or surgical time could predict facial function outcomes.

## Materials and methods

A retrospective study was carried out at the otorhinolaryngology department of a tertiary referral center, revising the local database and the medical charts of the patients with the approval of the Institutional Ethics Committee. The study included data on 157 patients who underwent surgical removal of a CPA tumor, with monitoring of facial nerve function by DSE and EMG. The facial nerve function was assessed immediately and at 12 days, 3 and 12 months postoperatively. The surgery was always performed by the same surgical team, between January 2013 and September 2020. Patients undergoing repeat surgery for the CPA tumor and those with alterations of the facial function before surgery (HB > 1) were excluded.

The pre-operative factors likely to present significant associations with facial nerve outcome (age, tumor size and pure tone average, PTA) were evaluated. Surgical time was also evaluated.

As regards the facial nerve function, the following intra-operative parameters likely to present significant associations with facial nerve outcome were evaluated: MST, proximal CMAP and the difference between proximal and distal CMAP (DPDC).

### Tumor size

All cases included in this study were diagnosed by brain MRI. Tumor size was recorded as the largest CPA diameter measured in the axial, coronal, or sagittal view: the portion of the tumor located inside the internal auditory canal (IAC)) was not counted. Tumor size was evaluated using the Tos and Thomson classification based on the largest extrameatal diameter only: intrameatal tumors, small tumors (1–10 mm extrameatal size), medium tumors (11–20 mm), large tumors (21–40 mm), and giant tumors (41 mm or larger) [15].

### House–Brackmann grading system

Facial nerve function was scored according to the House–Brackmann Grading System (Grades I–VI: Grade I, normal function; Grade VI, total palsy). The target disorder, poor facial nerve outcome, was defined as HB III–VI, while good facial nerve outcome was defined as HB I-II. Time-points of study for evaluation of the facial activity were short-term (12 days post-surgery) and long-term (1 year post-surgery) [16].

### Audiological tests

Pure-tone audiometry was performed to assess the air conduction hearing threshold in a range between 125 and 8000 Hz and in a range between 250 and 4000 Hz for bone conduction. The PTA of hearing threshold levels at a set of specified frequencies (500, 1000, 2000, 4000 Hz) was computed.

### Facial nerve monitoring

Intraoperative 2-channel EMG monitoring was performed with the NIM response 2.0 system (Medtronic Xomed Inc., Jacksonville, FL, USA) in all patients. Two paired bipolar subdermal electrodes were placed in the orbicularis oculi and in the oris muscles. Differences in impedance between electrodes remained less than 1 kΩ during the recordings on both channels. DES of the facial nerve was performed with a monopolar probe with a 0.5 mm tip. The stimulation applied was square waves of 100 ms duration with 4 Hz frequency. The monitor reports when a response greater than 1 µV occurs with a signal filter of 3.10 ms.

We consider the MST, the stimulus capable of producing 100 µV of CMAP response, recorded in at least one channel.

The stimulation protocol started with a 0.05 mA MST and was then decreased in intervals of 0.01 mA, up to a minimum stimulation of 0.01 mA or increased up to a maximum stimulation of 5.0 mA.

After tumor resection, the facial nerve proximal to the tumor, at the brain stem exit, was stimulated with the MST to obtain the proximal CMAP. The facial nerve was then stimulated with the same threshold at a point distal to the location of the tumor, in the IAC fundus, in order to obtain the distal CMAP. The peak-to-peak amplitude of each the proximal and distal CMAP was established and the difference was calculated (DPDC).

When MST could not be detected we considered the CMAP response to be 0, and so did not calculate the DPDC.

### Anesthesia management

Following the standard protocol for skull base surgery, anesthesia was induced with intravenous application of the sedative drug propofol (2–3 mg/kg), the opioid analgesic remifentanil (0.5 mcg/kg/3 min), and the skeletal muscle relaxant rocuronium (0.6 mg/kg). After intubation, rocuronium was omitted because of its interference with electrophysiological monitoring and mapping.

### Statistical analysis

Statistical analyses were performed using SPSS 25.0 (IBM SPSS Statistics, New York, USA). Continuous variables were presented as means with standard deviations and categorical variables were summarized with numbers and proportions. Data normality was assessed using the Kolmogorov–Smirnov test. The logistical regression was performed to analyse the preoperative clinical predictors that had impact on postoperative short- and long-term facial nerve outcome (poor or good). Spearman $\rho$ Index was used to obtain the correlation between size, duration of surgery, MST, CMAP, DPDC and HB.

Two-by-two tables were used to determine the sensitivity and specificity of MST, CMAP and DPDC for predicting short and long-term facial nerve outcomes. Sensitivity reflects the probability that the parameters to be within the defined zone, given that the patient has a good outcome. Specificity is the probability that parameters will be outside the defined zone, given that the patient has a poor outcome. Odds ratio and their corresponding 95% confidence intervals were calculated.

A “*p*” level of ≤ 0.05 was considered significant.

## Results

The study population consisted of 157 patients (54.7% male, 45.3% female), with a mean age of 51.9 years. Mean PTA before surgery was 61.7 dB and the mean surgical time was 8.1 h. In almost all cases the translabyrinthine approach was used (96.2%) with a final pathology result of VS in 93% and meningiomas in 7%. In some cases, a near total (35.7%) or a partial (3.8%) tumor resection was done to preserve the facial nerve, since the tumor was closely attached to it (Table 1).

**Table 1:** Study population

	Population *M* ± SD *N* (%)
Gender	
Male	86/157 (54.7%)
Female	71/157 (45.3%)
Age (years)	51.9 ± 13.1
Side	
Left	95/157 (60.5%)
Right	62/157 (39.5%)
PTA (dB)	61.7 ± 32.07
Size (mm)	
Extracanalicular	20.34 ± 10.7
Intracanalicular	8.18 ± 3.7
Duration of surgery (h)	8.11 ± 2.4
Approach	
Translabyrinthine	151/157 (96.2%)
Transotic	3/157 (1.9%)
Retrosigmoid	2/157(1.3%)
Medial fossa	1/157 (0.6%)
Tumor resection	
Total	95/157 (60.5%)
Near total	56/157 (35.7%)
Partial	6/157 (3.8%)
Anatomic pathology	
Vestibular Schwannoma	146/157 (93%)
Meningiomas	11/157 (7%)
Tumors associated with NF2	5/157 (3.1%)

*N* number, *%* percentage, *M* media, *SD* standard deviation, *PTA* pure tone average, *NF2* neurofibromatosis type 2

Most of the tumors were medium or large according to the Tos and Thomsen Classification 124/157 (79%). Only tumors which, after careful follow up, were seen to have increased in size or tumors which reached the brainstem were operated (Table 1 Supplemental material).

**Fig. 1:**
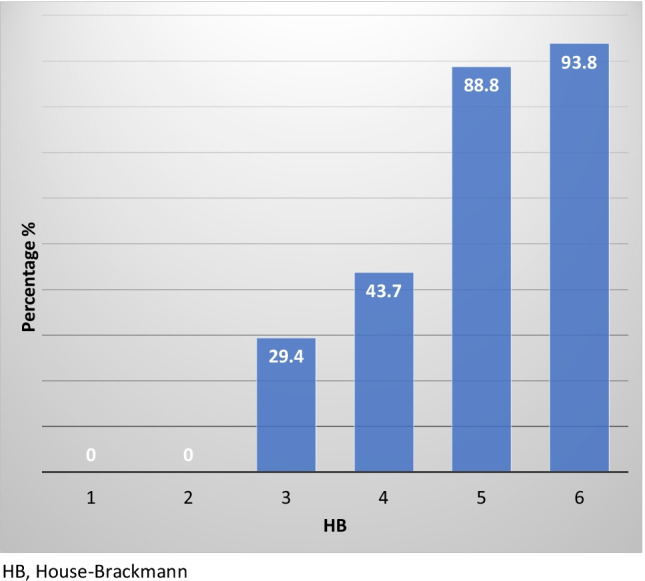
Logistic regression model: Immediate post-operative HB and probability of long-term (1 year) negative outcome. *HB* House–Brackmann

Short-term facial outcome was good in 44% of cases (HB I, 31%; HB II, 13%), poor outcome was obtained in 56% (HB III, 12%; HB IV, 12%; HBV 23%; HB VI, 9%) (Fig. 1. Supplemental material).

**Fig. 2:**
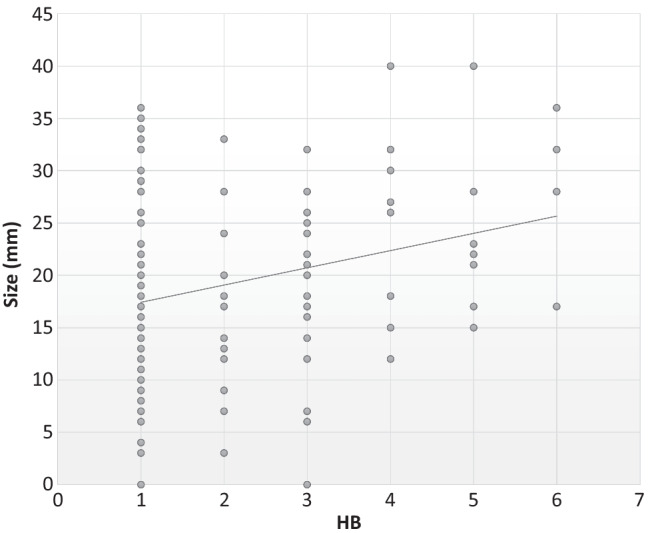
The size increase is correlated with postoperative House–Brackmann grade in the long term ($\rho$ 0.18, *p* = 0.03). The diagonal line represents a linear regression model to the data. The risk of an HB 3 or more begins with a size of 20.2 mm

Long-term facial outcome was good in 61.1% of cases (HB I, 50.4%; HB II, 10.7%), and poor in 38.9% (HB III, 18.5%; HB IV, 9.5%; HB V, 7%; HB VI, 3.9%) (Fig. 2. Supplemental material). The worst long-term facial outcomes were obtained in giant and large tumors (Fig. 3. Supplemental material).

Logistical regression was applied to analyse the preoperative clinical predictors that had an impact on postoperative short- and long-term facial nerve outcome. The factors with a statistically significant association were size, MST, CMAP for short- and long-term outcome (Table 2), and duration of surgery and DPDC only for short-term outcome (Table 2 Supplemental material). No significant association with age and PTA (*p* > 0.05) was found.

**Table 2:** Logistical regression analysis of preoperative clinical predictors with regard to their impact on postoperative facial function long-term outcomes (1 year)

	Odds Ratio	95% CI	*p* value
Age	0.99	0.96–1.02	0.56
Size	1.06	1–1.07	0.04
Duration of surgery	1.16	0.99–1.37	0.06
PTA	1.01	0.99–1.01	0.55
MST	114.12	4.02–3475.2	< 0.01
CMAP	0.99	0.99–0.99	< 0.01
Difference proximal and distal CMAP	0.99	0.99–1	0.14

*PTA* pure tone average, *MST* minimum stimulation threshold, *CMAP* compound muscle action potential, *CI* confidence Interval

The logistical regression model was also used to associate immediate post-operative HB and the probability of a long-term (1 year) negative outcome (Fig. 1). All patients with an HB I-II on immediate post-surgery examination maintained a good facial outcome after 1 year of follow-up. A postoperative HB V or VI was correlated with a poor outcome in 88.8% and 93.8% of cases. Thus, the model demonstrated that a higher grade of immediate facial paralysis corresponds to a higher risk of poor outcome after one year.

Regarding tumor size, positive correlations with short- (*r* = 0.25, *p* = 0.01) and long-term (*r* = 0.18, *p* = 0.03) HB grade were found (Table 3  Supplemental material). A linear regression model showed that a risk of a HB 3 or more in the long term was correlated with a mean size of 20.2 mm (Fig. 2).

**Table 3:** Correlation using Spearman $\rho$ Index between MST, proximal CMAP, proximal and distal CMAP difference and HB

	MST *r* (*p* value)	Proximal CMAP *r* (*p* value)	Difference proximal and distal CMAP *r* (*p* value)
HB post-surgery	0.51 (< 0.01)	– 0.71 (< 0.01)	– 0.45 (< 0.01)
HB after 12 days	0.45 (< 0.01)	– 0.66 (< 0.01)	– 0.36 (< 0.01)
HB after 3 months	0.47 (< 0.01)	– 0.63 (< 0.01)	– 0.21 (0.13)
HB after 1 year	0.40 (< 0.01)	– 0.62 (< 0.01)	0.20(0.16)

*MST* minimum stimulation threshold, *CMAP* compound muscle action potential, *HB* House–Brackmann, *r* Spearman $\rho$ Index

As for surgical time, a positive correlation with short-term (*r* = 0.26, *p* < 0.01) HB grade was found (Table 3 Supplemental material). The linear regression model showed that a risk of an HB 3 or more, in the short term, was correlated with an operation lasting more than 7.8 h (Fig. 4 Supplemental material).

Regarding the intra-operative DES during facial nerve monitoring, a positive correlation between MST and short (*r* = 0.45, *p* < 0.01) and long-term (*r* = 0.40, *p* < 0.01) HB grade was found. A negative correlation was calculated between proximal CMAP and short- (*r* = − 0.71, *p* < 0.01) and long-term (*r* = − 0.62, *p* < 0.01) HB grade. A negative correlation between the DPDC and only the short-term (*r* = − 0.36; *p* < 0.01) HB grade (Table 3) was also found.

The sensitivity and specificity of the parameters of the intra-operative DES were calculated for both short-term (Table 4 Supplemental material) and long-term poor facial outcome (Table 4). When an MST was not detected during the DES, and consequently not even one CMAP, the specificity was 100% for both short- and long-term poor outcome.

**Table 4:** Long-term sensitivity and specificity of MST, proximal CMAP and proximal and distal CMAP difference in facial outcome

	Sensitivity (CI 95%)	Specificity (CI 95%)
MTS cut-off (mA)		
Not detected	33% (0.2–0.49)	100% (0.93–1)
0.1	62% (0.46–0.75)	73% (0.61–0.82)
0.05	91% (0.77–0.97)	21% (0.13–0.33)
CMAP (µV)		
< 500	97% (0.80–0.99)	30% (0.16–0.47)
< 200	73% (0.53–0.87)	73% (0.55–0.85)
Difference proximal and distal CMAP (µV)		
< 0	79% (0.48–0.94)	38% (0.23–0.55)

*MST* minimum stimulation threshold, *CMAP* compound muscle action potential, *CI* confidence Interval

Using 0.10 mA MST as a cut-off point, a sensitivity of 0.55 (95% CI 0.42–0.67) and a specificity of 0.75 (95% CI 0.61–0.85) for short-term facial nerve activity prediction was detected. For long-term prognosis, sensitivity and specificity were 0.62 (95% CI 0.46–0.75) and 0.73 (95% CI 0.61–0.82).

Using an MST of 0.05 mA, for short-term function, a sensitivity of 0.94 (95% CI 0.83–0.97) and a specificity of 0.28 were calculated. For long-term function, sensitivity was 0.91 (95% CI 0.77–0.97) and specificity 0.21.

When used to predict short-term function, CMAP 500 µV had a sensitivity of 0.95 (95% CI 0.82–0.99) and a specificity of 0.37. To predict long-term function, sensitivity was 0.97 (95% CI 0.80–0.99) and specificity 0.3.

The most significant results were obtained using a CMAP cut-off < 200 µV. For short-term function, a sensitivity of 0.62 (95% CI 0.46–0.76) and a specificity of 0.79 (95% CI 0.57–0.92) were calculated. For long-term prediction, sensitivity and specificity were 0.73 (95% CI 0.53–0.87) and 0.73 (95% CI 0.55–0.85), respectively.

Analysis of the logistical regression and the correlation data showed DPDC to be useful only for short-term outcome. Setting a DPDC cut-off < 0 (distal stimulation higher than the proximal one), a sensitivity of 0.89 (0.7–0.9) and a specificity of 0.58 (0.37–0.77) were obtained.

## Discussion

Today, facial monitoring is an essential instrument in CPA surgery. It helps to prevent unintended injury to the facial nerve, as DES allows the recognition of the nerve and enables more careful tumor dissection. Even at experienced centers and with the use of facial nerve monitoring, the facial nerve is at risk in CPA tumor surgery. Therefore, a significant number of patients will have delayed or permanent facial nerve dysfunction even though the nerve is preserved. The incidence of long-term facial dysfunction after VS treatment varies widely, from 4.8 to 41% in previous publications, with a mean of approximately 19% [17].

In addition, the ability to accurately predict short- and long-term facial nerve outcomes remains unclear, especially in large tumors [1].

Several studies have shown that immediate post-operative preservation of facial nerve function (HB I–II) is an excellent prognostic indicator of a good facial nerve outcome. Patients with immediate facial dysfunction are at a greater risk of poor recovery. In our study, 100% of patients who had intact eye closure on immediate post-surgery examination remained House–Brackmann grade I or II after one year of follow-up. A postoperative HB V or VI was correlated with poor outcome in 88.8% and 93.8% of cases, respectively, in agreement with other articles that showed the positive prognostic value of immediate postoperative HB to be between 94 and 98% [18–20]. In any case, patients with poor immediate outcome (particularly HB III) may improve with time [21].

With regard to tumor size, a positive correlation with short- and long-term facial outcome was found, with a risk of HB III or more in tumors larger than 20.2 mm. Rinaldi et al. reported an increased incidence of facial deficit in patients with stage IV tumors in the Zini-Magnan classification (> 2 cm) [22] either in the immediate postoperative period or 1 year after surgery [23]. Other authors have reported that the smaller the tumor, the better the subsequent facial function. Especially in the case of larger tumors, this association may be related to a pre-existing nerve weakness (due to the compression caused by the tumor) and to increased number of difficult surgical maneuvers during the dissection of the tumor from the nerve [24, 25].

Regarding the duration of the intervention, a positive correlation was found with short-term facial outcome, with an increased risk of HB grade III or above with a duration longer than 7.8 h. Zaouche et al. stated that the case of hemorrhagic tumors, adhesion between the tumor and the facial nerve, the cerebellum, or the brainstem lengthened the procedure and worsened the facial outcome [26].

As regards MST, a positive correlation was found with facial outcome in both the short and the long term. The present study used 0.05 mA and 0.1 mA cut-offs, obtaining a high sensitivity (91% short-term and 95% long-term) for 0.05 mA and a good specificity (75% short-term and 73% long term) and sensitivity (62% long-term) for 0.1 mA, in agreement with other recently published studies. Huang et al. found the sensitivity and specificity (12 months postoperatively) of the “low current” of 0.05 mA for predicting facial function outcome were 87.2 and 48.0%, respectively. In this study, for patients with HB IV in the first month post-surgery, the recovery rate in the group with MST 0.05 mA was significantly higher than that of the control group with a higher MST [27]. In the study by Schmitt et al., using an MST of 0.1 mA, the sensitivity and specificity for long-term facial outcome were 94% and 12%, respectively [28]. The most recent meta-analysis, by Quimby et al., reports that an MST cut-off of 0.10 mA had a sensitivity of 0.76 (95% CI 0.53–0.90) and a specificity of 0.68 (95% CI 0.42–0.87), and an MST cut-off of 0.05 mA had a sensitivity of 0.73 (95% CI 0.58–0.84) and a specificity of 0.74 (95% CI 0.59–0.85) for facial function in the long term [29].

A negative correlation was found between proximal CMAP and facial outcome in both the short and the long term. CMAP cut-offs of 500 µV and 200 µV were used, obtaining a high sensitivity (95% short-term and 97% long-term) for 500 µV and a good sensitivity (62% short-term and 73% long-term) and specificity (79% short-term and 73% long-term) for 200 µV. Using a cut-off of 500 µV, Magliulo et al. reported the sensitivity and specificity for short-term facial outcome to be 83% and 65%, respectively [30]. Neff et al. used logistic regression modelling to find a significant relation between MST and CMAP, which was able to compare between good and poor long-term results. The same authors found that MST 0.05 mA and CMAP 240 mV had 88% sensitivity and 85% specificity for good long-term facial prognosis [31]. In their meta-analysis, Quimby et al. reported a sensitivity of 0.87 (95% CI 0.78–0.93) and a specificity 0.59 (95% CI 0.50– 0.68) [29] for CMAP 500 mV to predict short-term function. The data regarding the sensitivity of CMAP 500 µV observed in the present study corroborate the results of recent reports. As regards the cut-off of 200 µV, with which we obtained excellent predictive results, more studies are needed for comparison.

With respect to the evaluation of the difference between proximal and distal CMAP, the cut-off < 0 used in the present study obtained a sensitivity of 79% for short-term outcomes. In the recent literature there are few studies on the role of DPDC as a prognostic factor of postsurgical facial outcomes, but two articles valued a proximal-to-distal CMAP amplitude ratio of 2:3, and another evaluated a ratio of 1.0. A ratio of 2:3 was associated with a sensitivity for short-term function of 0.67–0.93 and a specificity of 0.80 to 0.84. A ratio of 1.00 had sensitivities and specificities of 0.2 and 0.89 for short-term function and 0.25 and 0.88 for long-term function, respectively [32–34].

Electromyography monitoring of the facial nerve is based on the measurement of CMAP elicited by DES of the nerve proximally, close to the brain stem, after tumor resection. However, EMG could only be obtained from the point of nerve stimulation to the facial muscles, without tracking the entire full-length pathway. Therefore, the recording of the CMAP may reflect a stimulation of the distal part even in the case the facial is damaged proximally. Indeed, recent studies suggest that the signals generated by the facial nerve near the brainstem are the most significant and representative [35–37].

In some cases, a patient with a low MST and a high CMAP may develop a more severe facial dysfunction than expected, due to the event of delayed-onset facial nerve paresis. Delayed-onset facial nerve dysfunction is a deterioration of at least one HB Grade between 3 and 30 days after surgery. The hypothesis proposed is that there is a latent reactivation of a dormant neurotrophic virus harbored in the facial nerve which can be seen in between 4.8 and 41% of patients undergoing VS surgery. This process is not predicted by intraoperative facial monitoring because it postdates the surgery, and it may be a factor leading to a poorer than expected facial nerve outcome [38–40].

The present study presents some limitations. First, it is a single-center retrospective study with a low number of cases. Second, the grading of the facial function was evaluated by the same medical team in the same department, and the raters were not blinded to the DES parameters, which might have increased the bias. Third, monopolar stimulation was used, although it’s been suggested that bipolar stimulation decreases the risk of false-negative responses and seems to be more accurate, to avoid this limitation monopolar stimulation was used with minimal threshold stimulation (MTS) of 0.05–0.1 mA, when identifying facial nerve during tumor removal.

To resolve some of these limitations, a larger prospective blinded case-cohort study is needed to fully evaluate the process of facial function recovery after VS removal.

## Conclusion

Facial nerve paresis or paralysis after VS surgery continues to be a significant source of morbidity. Parameters obtained by DES of the facial nerve after tumor resection should be an important advantage for prognosticating facial functional results in CPA surgery. Immediate HB, tumor size, MST, and proximal CMAP are correlated with long-term facial outcome. Based on the different cut-offs analysed, it is possible to predict the outcome of facial activity with considerable accuracy.

Refining predictive accuracy helps surgeons to meet patients’ perspectives and to elaborate an intervention program in order to plan facial rehabilitation or set up a surgical anastomosis.

## Supplementary Information

Below is the link to the electronic supplementary material.

Supplementary file1 (DOCX 324 KB)
