# Supplementary material for: Pre- and intra-operative prognostic factors of facial nerve function in cerebellopontine angle surgery
Source: Eur Arch Otorhinolaryngol. 2022 Jul 30;280(3):1055–62. doi: 10.1007/s00405-022-07556-8 (PMC9899719; doi:10.1007/s00405-022-07556-8)
Supplement: Supplementary file 1 — Supplementary file1 (DOCX 324 KB) [file 405_2022_7556_MOESM1_ESM.docx]

**Table 1.** Tos and Thomsen Classification applied to the case series

| **Size** | | **N** | **%** |
| --- | --- | --- | --- |
| Small (< o = 1 cm) | | 27 | 17.2% |
| Medium (1-2.5 cm) | | 81 | 51.6% |
| Large (2.5-4 cm) | | 43 | 27.4% |
| Giant (> 4 cm) | | 6 | 3.8% |
| Total |  | 157 | 100% |

N, number; %, percentage;

**Table 2.** Logistical regression analysis of preoperative clinical predictors with regard to their impact on postoperative facial function **short-term outcomes** (12 days)

|  | **Odds Ratio** | **95%CI** | **p value** |
| --- | --- | --- | --- |
| Age | 1.01 | 0.98-1.03 | 0.41 |
| **Size** | 1.05 | 1.01-1.09 | <0.01 |
| **Duration of surgery** | 1.24 | 1.05-1.45 | <0.01 |
| PTA | 1.01 | 0.99-1.01 | 0.35 |
| **MST** | 76.34 | 2.13-2726.7 | <0.01 |
| **CMAP** | 0.99 | 0.99-0.99 | <0.01 |
| **Difference proximal and distal CMAP** | 0.99 | 0.99-0.99 | <0.01 |

PTA, Pure tone average; MST, minimum stimulation threshold; CMAP, compound muscle action potential; CI, Confidence Interval

**Table 3**. Correlation using Spearman rho Index between size (mm), duration of surgery (hours) and HB

|  | **Size**  **r (p value)** | **Duration of surgery**  **r (p value)** |
| --- | --- | --- |
| HB immediate post-surgery | 0.25 (0.01) | 0.33 (<0.01) |
| HB after 12 days | 0.25 (0.01) | 0.26 (<0.01) |
| HB after 3 months | 0.21 (0.01) | 0.20 (0.02) |
| HB after 1 year | 0.18 (0.03) | 0.03 (0.56) |

HB, House-Brackmann

**Table 4.** Short-term sensitivity and specificity of MST, proximal CMAP and proximal and distal CMAP difference in facial outcome

|  | **Sensitivity (95%CI)** | **Specificity (95%CI)** |
| --- | --- | --- |
| MTS cut-off (mA) |  |  |
| Not detected | 23% (0.14-0.36) | 100% (0.92-1) |
| 0.1 | 55% (0.42-0.67) | 75% (0.61-0.85) |
| 0.05 | 94% (0.83-0.97) | 28% (0.17-0.42) |
| CMAP (µV) |  |  |
| <500 | 95% (0.82-0.99) | 37% (0.19-0.59) |
| <200 | 62% (0.46-0.76) | 79% (0.57-0.92) |
| Difference proximal and distal CMAP (µV) |  |  |
| <0 | 89% (0.7-0.97) | 58% (0.37-0.77) |

MST, minimum stimulation threshold; CMAP, compound muscle action potential; CI, Confidence Interval

**Figure 1**. **A** Percentage of HB (I-VI) after 12 days of follow-up; **B** Percentage of good (HB I-II) and poor (HB III-VI) outcome after 12 days.


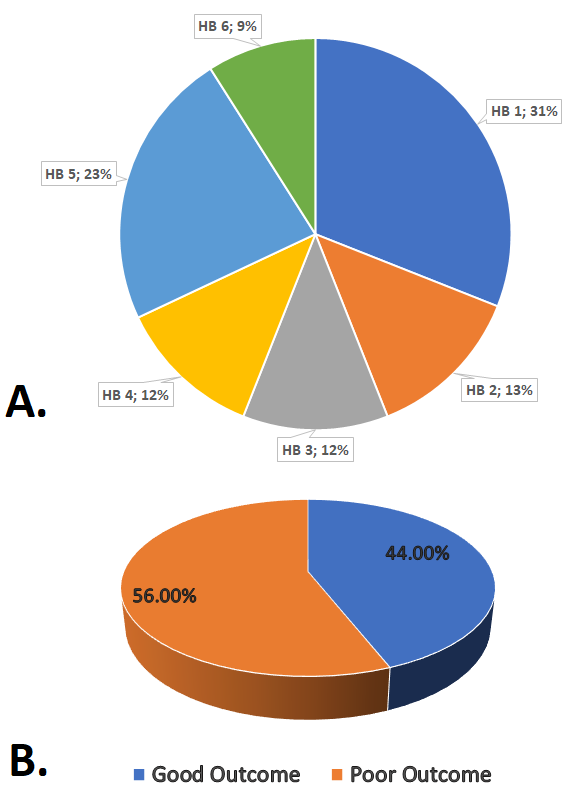


**Figure 2. A** Percentage of HB (I-VI) after 1 year of follow-up; **B** Percentage of good (HB I-II) and poor (HB III-VI) outcome after 1 year.

**
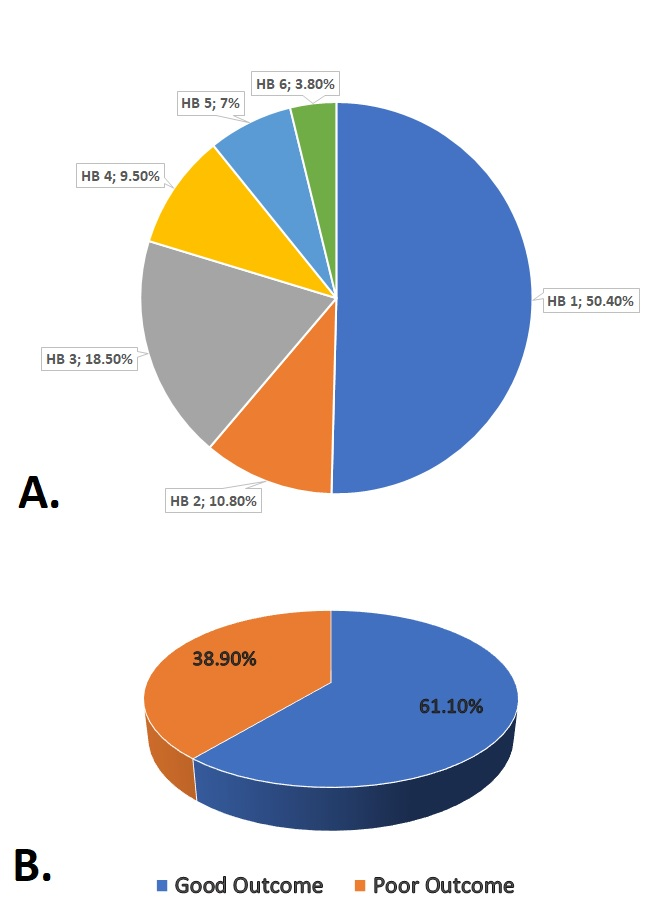
**

**Figure 3**. Percentage of HB (I-VI) after 1 year of follow-up, related to Tos and Thomsen classification


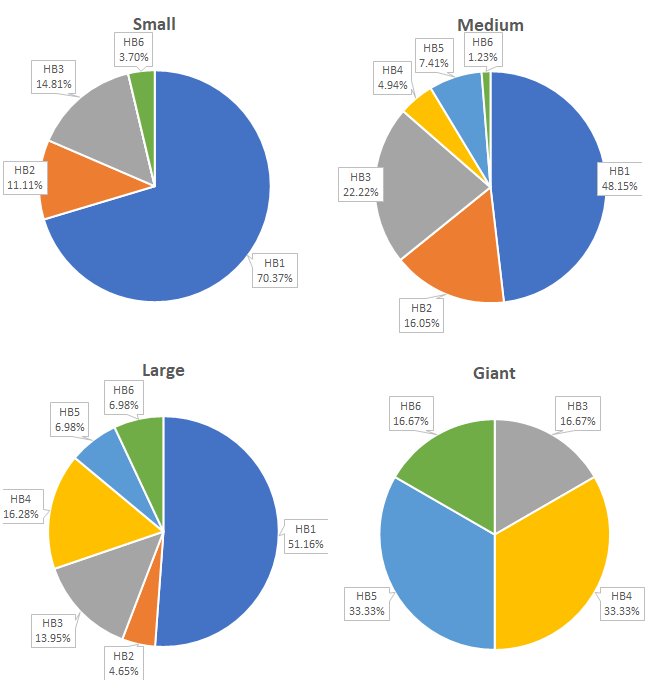


**Figure 4.** The increase in surgical time is correlated with the postoperative House-Brackmann grade in the short term (rho 0.26, p <0.01). The diagonal line represents a linear regression model to the data. The risk of HB 3 or more begins with a surgical time of more than 7.8 hours.
